# MiR-328 targeting PIM-1 inhibits proliferation and migration of pulmonary arterial smooth muscle cells in PDGFBB signaling pathway

**DOI:** 10.18632/oncotarget.10714

**Published:** 2016-07-19

**Authors:** Zhengjiang Qian, Limin Zhang, Jidong Chen, Yanjiao Li, Kang Kang, Junle Qu, Zhiwei Wang, Yujia Zhai, Li Li, Deming Gou

**Affiliations:** ^1^ Shenzhen Key Laboratory of Microbial Genetic Engineering, College of Life Sciences and Oceanography, Shenzhen University, Shenzhen, Guangdong, 518060, China; ^2^ Key Laboratory of Optoelectronic Devices and Systems of Ministry of Education, College of Optoelectronic Engineering, Shenzhen University, Shenzhen, Guangdong, 518060, China; ^3^ Department of Biochemistry and Molecular Biology, School of Basic Medical Sciences, Shenzhen University, Shenzhen, Guangdong, 518000, China

**Keywords:** miR-328, PASMCs, PDGF pathway, PIM-1

## Abstract

MicroRNAs (miRNAs) have been recognized to mediate PDGF-induced cell dysregulation, but their exact functions remain to be elucidated. By using a sensitive S-Poly(T) Plus qRT-PCR method, the expression profiling of 1,078 miRNAs were investigated in pulmonary artery smooth muscle cells (PASMCs) with or without PDGFBB stimulation. MiR-328 was found as a prominent down-regulated miRNA, displaying a specific dose- and time-dependent downregulation upon PDGFBB exposure. Functional analyses revealed that miR-328 could inhibit PASMCs proliferation and migration both with and without PDGFBB treatment. The Ser/Thr-protein kinase-1 (PIM-1) was identified as a direct target of miR-328, and functionally confirmed by a rescue experiment. In addition, the decrease of miR-328 by PDGFBB might be due to the increased expression of DNA methylation transferase 1 (DNMT1) and DNA methylation. Finally, serum miR-328 level was downregulated in PAH patients associated with congenital heart disease (CHD- PAH). Overall, this study provides critical insight into fundamental regulatory mechanism of miR-328 in PDGFBB-activited PASMCs via targeting PIM- 1, and implies the potential of serum miR-328 level as a circulating biomarker for CHD- PAH diagnosis.

## INTRODUCTION

Pulmonary arterial smooth muscle cells (PASMCs) constitute a major cell layer of pulmonary vascular and play a critical role in maintaining vessel tone and blood pressure. Under normal condition, PASMCs are retained in a non-proliferative and non-migratory state. The quiescent state, however, can be disturbed by varieties of stimuli, leading to an increase in the pulmonary artery pressure and vascular remodeling of pulmonary vessels [1, 2]. It has been abundantly documented that aberrant proliferation and migration of PASMCs were key pathological processes in the genesis and development of pulmonary arterial hypertension (PAH), a progressive and life-threatening disease [1, 3, 4]. Thus, intensive attention was paid to the dysfunctional PASMCs [2].

In addition to genetic, epigenetic and environmental factors [4–6], the state of vascular smooth muscle cells (VSMCs) can also be affected by numerous growth factors and cytokines, such as platelet-derived growth factor BB (PDGFBB), fibroblast growth factor (FGF), transforming growth factor beta (TGF-β), angiotensin II (Ang II) and endothelin-1 (ET-1) [1, 2]. Among them, PDGFBB is known as a potent mitogen and chemoattractant for VSMCs. Via the activation of downstream transcriptional factors and molecular signaling pathway, PDGF can trigger various biological processes and the occurrence of many vascular diseases, including PAH. Accordingly, blocking the PDGF signals could efficiently prevent the dysregulation of PASMCs, and subsequently attenuate the progression and symptom of PAH [7–9]. Therefore, the discovery of novel regulatory molecules in PGDF signaling pathway are of great scientific and therapeutic interest.

There is now accumulating evidence suggesting that microRNAs (miRNAs), a class of small endogenous non-coding RNA, can be essential in regulating PASMCs function [10–16]. MiRNAs are known to negatively regulate gene expression by mainly interacting with the 3′-untranslated region (UTR) of their target mRNAs [10–12]. Recent studies indicate that differentially expressed miRNAs upon PDGF stimulation play pivotal roles in driving cell dysfunction. For instance, miR-221 that is upregulated by PDGF, increases VSMCs proliferation and migration by suppressing p27Kipl [17]. Through the ERK1/2 Pathway, PDGF-induced miR-136 promotes VSMCs proliferation via targeting PPP2R2A (protein phosphatase 2, regulatory subunit B, alpha) [18]. By contrast, the decrease of miR-638 expression by PDGFBB suppresses the VSMCs proliferation and migration through targeting NOR1 (Neuron-derived orphan receptor-1) [19]. Despite promising progresses have been achieved in discovering the function of miRNA in response to PDGF, the cellular and molecular mechanisms involved in these processes are still largely unexplored.

In this study, based on the expression profile analyses of 1,078 miRNAs, we investigated the role of miR-328 in PDGFBB-stimulated PASMCs, and measured serum miR-328 level in PAH patients associated with congenital heart disease (CHD-PAH). Our results demonstrated that miR-328 inhibited the proliferation and migration of PASMCs through targeting PIM-1, and the specific downregulation of miR-328 by PDGFBB might attribute to the increase of DNMT1 expression and DNA methylation. Moreover, we showed that serum miR-328 decreased in CHD-PAH, supporting its potential as a circulating biomarker for CHD-PAH diagnosis.

## RESULTS

### MiR-328 expression is downregulated by PDGFBB in PASMCs

**Figure 1:**
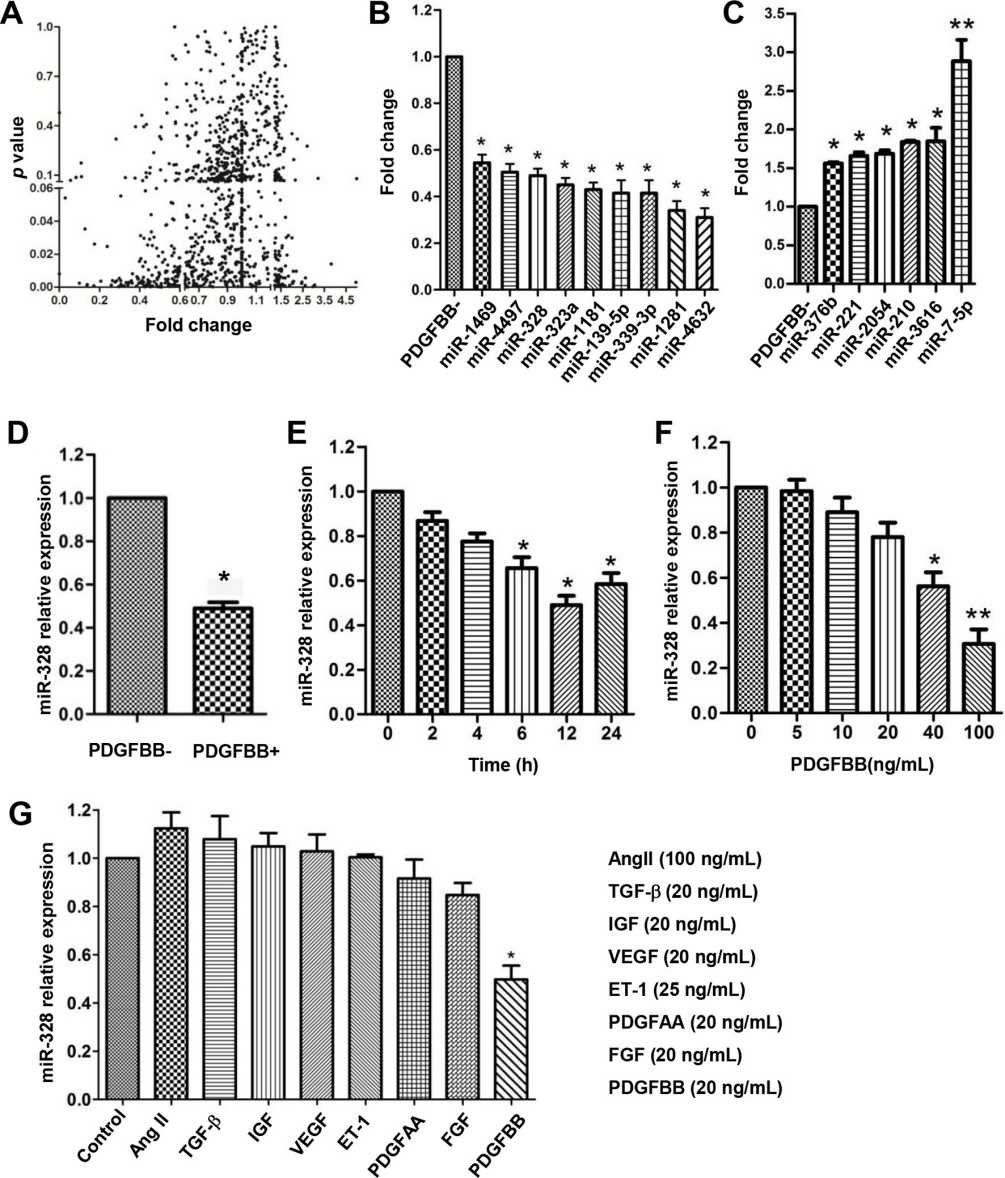
miR-328 is downregulated in PDGFBB-stimulated PASMCs Human pulmonary artery smooth muscle cells (PASMCs) were starved for 12 h in starvation condition (0.2% FBS) before treating with PDGFBB (20 ng/mL) for 24 h. The differential expression of 1,087 miRNAs were profiled using a sensitive S-Poly(T) Plus qRT-PCR method (**A**), leading to the identification of 9 downregulated (**B**) and 6 upregulated miRNAs (**C**). The downregulation of miR-328 was further confirmed in rat PASMCs (**D**), displaying a time- and dose-dependent manner upon PDGFBB treatment (**E** and **F**). MiR-328 expression was specifically inhibited by PDGFBB rather than other growth factors including AngII, TGF-β, IGF, VEGF, ET-1, PDGFAA and FGF at concentrations indicated (**G**). Data are expressed as means ± SD with at least three independent experiments. *p < 0.05, **p < 0.01 compared to control without PDGFBB treatment (PDGFBB-).

To identify differentially expressed miRNAs in PDGFBB pathway, a genome wide miRNAs expression profiling analysis was performed in human PASMCs after 24 h of PDGFBB treatment (Figure 1A). Among 1,078-profiled miRNAs, a total of 15 significantly changed miRNAs were identified, including 9 down-regulated and 6 up-regulated miRNAs (Figure 1B and 1C). The downregulation of miR-328 by PDGFBB was further confirmed in rat PASMCs (Figure 1D), displaying a time- and dose-dependent manner (Figure 1E and 1F). Interestingly, the expression of miR-328 was specifically inhibited by the treatment of PDGFBB rather than other growth factors, such as AngII, TGF-b, IGF, VEGF, ET1, PDGFAA and FGF (Figure 1G).

### MiR-328 inhibits PASMCs proliferation and migration

**Figure 2:**
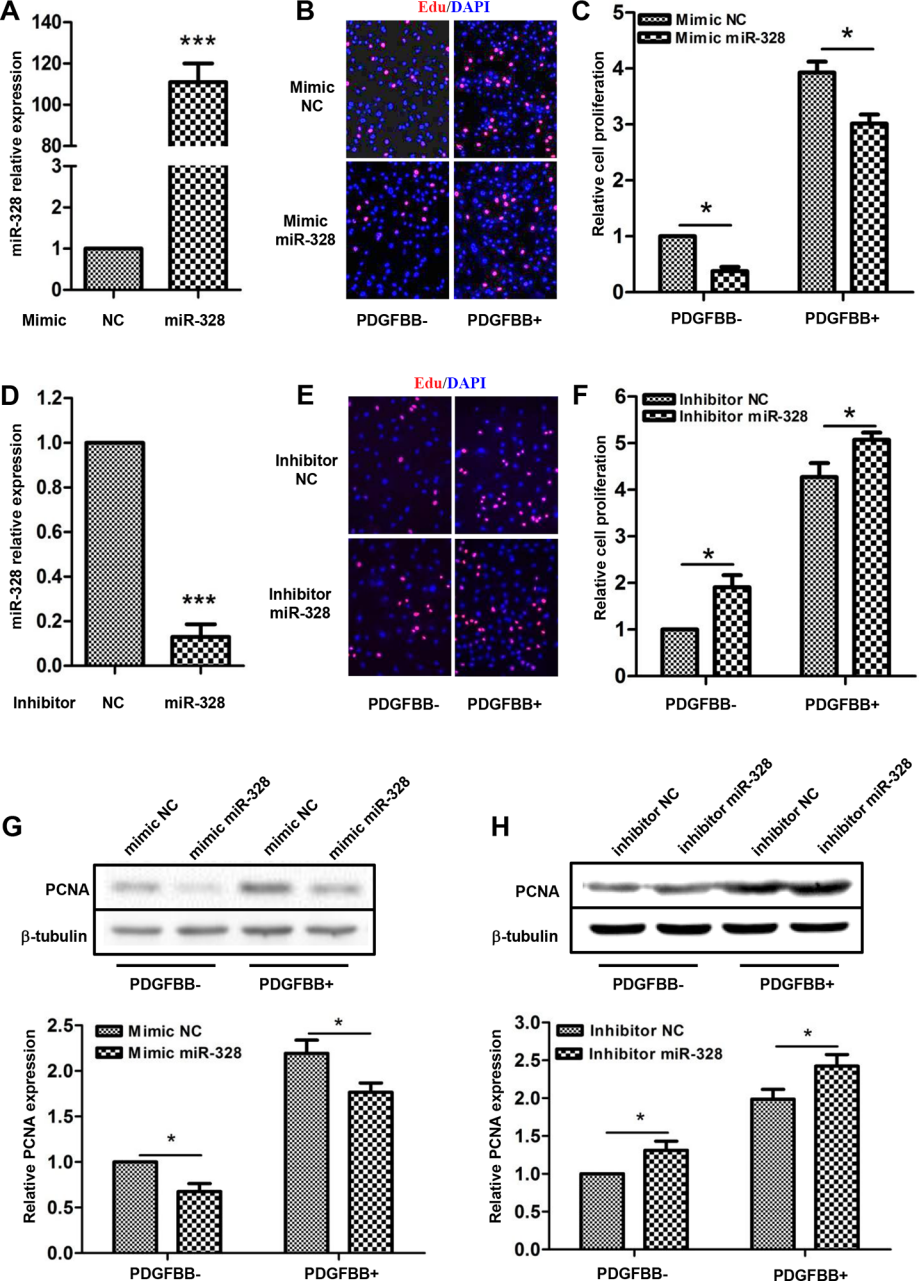
miR-328 inhibits PASMCs proliferation The relative expression of miR-328 was analyzed in PASMCs transfected with miR-328 mimic or inhibitor by quantitative PCR, as compared with their negative control (NC) (**A** and **D**); ***P < 0.001 compared to NC. The proliferation of PASMCs was measured by EdU incorporation assays. Representative images of EdU labeling showing proliferation of PASMCs transfected with either miR-328 mimic (**B**) or inhibitor (**E**) with and without PDGFBB stimulation. The quantifications were shown to better view the difference between different treatments (**C** and **F**). The protein level of PCNA was detected by Western blotting, and b-tubulin was used as internal control. Representative results of immunoblots and their quantifications were shown (**D** and **H**). Data are presented as means ± SD with three independent experiments; *p < 0.05 compared to control without PDGFBB treatment (PDGFBB-).

Mimic or inhibitor miR-328 was transfected into PASMCs to investigate whether miR-328 mediates cell proliferation. Accordingly, miR-328 expression increased by over 100 folds or decreased by 90% through these two transfection events, respectively (Figure 2A and 2D). As compared with mimic negative control (NC), transfection of mimic miR-328 significantly inhibited PASMCs proliferation both with and without PDGFBB stimulation (Figure 2B and 2C). By contrast, inhibition of miR-328 promoted the proliferation of PASMCs (Figure 2E and 2F). In parallel, the effects of miR-328 on PASMCs proliferation was further evaluated by determining the expression of the proliferating cell nuclear antigen (PCNA). Consistently, mimic miR-328 decreased while its inhibitor increased PCNA expression, as compared to their NC, respectively (Figure 2G and 2H).

**Figure 3:**
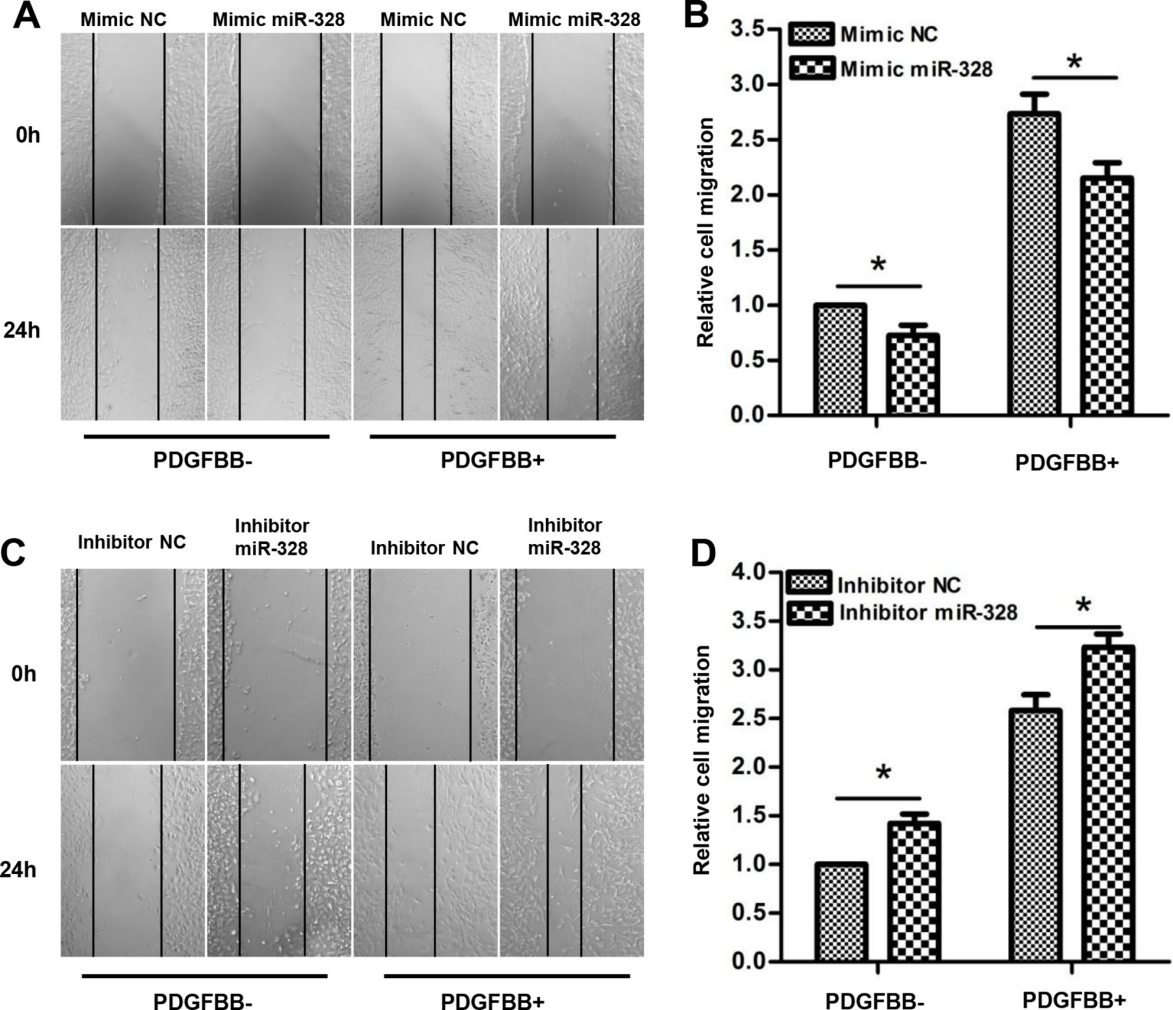
miR-328 inhibits PASMCs migration After 24 h of transfection of mimic miR-328/mimic NC or inhibitor miR-328/inhibitor NC, PASMCs were starved for 12 h. PASMCs migration was determined after 24h of PDGFBB treatment by wound-healing assay. Representative images of wound-healing assay showing migration of PASMCs transfected with miR-328 mimic (**A**) or inhibitor (**C**) with and without PDGFBB treatment, and their respective quantifications (**B** and **D**). Results are shown as means ± SD of three independent experiments. *p < 0.05 compared to mimic/inhibitor NC without PDGFBB treatment (PDGFBB-).

In addition, the effects of miR-328 on PASMCs migration were measured using wound healing assays. As shown in (Figure 3A and 3B), transfection of mimic miR-328 significantly suppressed PASMCs migration both in the absence and presence of PDGFBB. On the contrary, PASMCs transfected with miR-328 inhibitor exhibited greater migratory rate as compared to inhibitor NC (Figure 3C and 3D). Altogether, these data suggested that miR-328 could inhibit the proliferation and migration of PASMCs with and without PDGFBB treatment.

### PIM-1 is a direct target of miR-328

**Figure 4:**
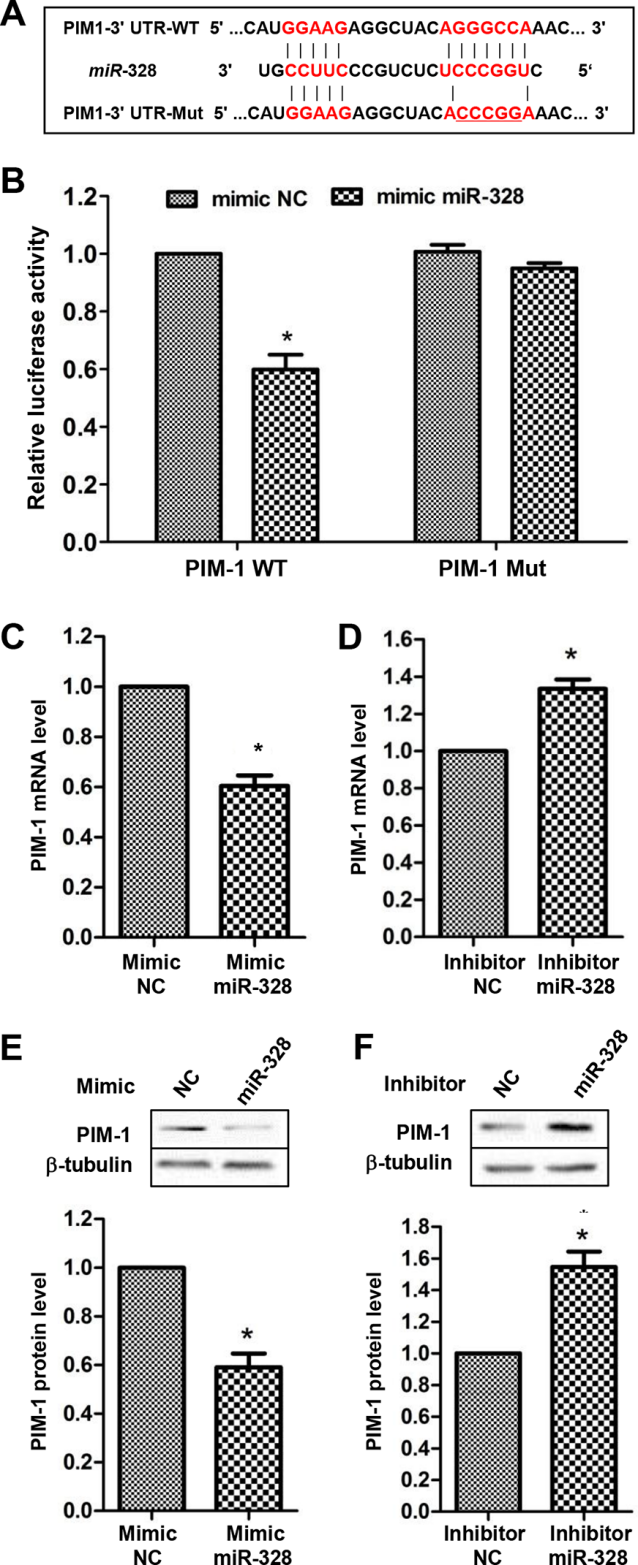
PIM-1 is a direct target of miR-328 A diagram of miR-328 putative binding site in human PIM-1 3′-UTR and alignment of PIM-1 wild-type (PIM-1 WT) and mutated 3′-UTR binding site (PIM-1 Mut) of miR-328 (**A**). The five mutated nucleotides are underlined. PIM-1 WT or PIM-1 Mut construct was cotransfected with mimic miR-328 or mimic negative control (mimic NC) in HEK 293 cells, and luciferase reporter assay was performed to assess interaction between miR-328 and 3′-UTR of PIM-1 (**B**), *p < 0.05 compared to cotransfection of mimic NC and PIM-1 WT vector. PASMCs were transfected with mimic miR-328/mimic NC or inhibitor miR-328/inhibitor NC for 48 h, and the mRNA and protein level of PIM-1 were measured by qRT–PCR (**C** and **D** and western blot (**E** and **F**). Results are shown as means ± SD of three independent experiments. *p < 0.05 compared to mimic/inhibitor NC.

Via a search on Targetscan database (http://www.targetscan.org/) and previous literatures, five genes including *PIM-1*, *MMP2*, *BSG*, *PAX6* and *ABCG2* were found as candidate targets of miR-328. Base on luciferase activity assays, *PIM-1* was chosen for further investigation. The luciferase activity was significantly reduced by co-transfection of mimic miR-328 with *PIM-1* 3′UTR reporter vector. By contrast, mutation of miR-328 binding site in the 3′-UTR of *PIM-1* caused a complete restoration of luciferase activity (Figure 4A and 4B). Moreover, overexpressing miR-328 in PASMCs markedly decreased (Figure 4C and 4E), whereas suppressing miR-328 increased the expression of PIM-1 at both mRNA and protein levels (Figure 4D and 4F). Collectively, these results showed that PIM-1 is a direct target of miR-328 in PASMCs.

### PIM-1 knockdown rescues proliferating PASMCs by inhibition of miR-328

**Figure 5:**
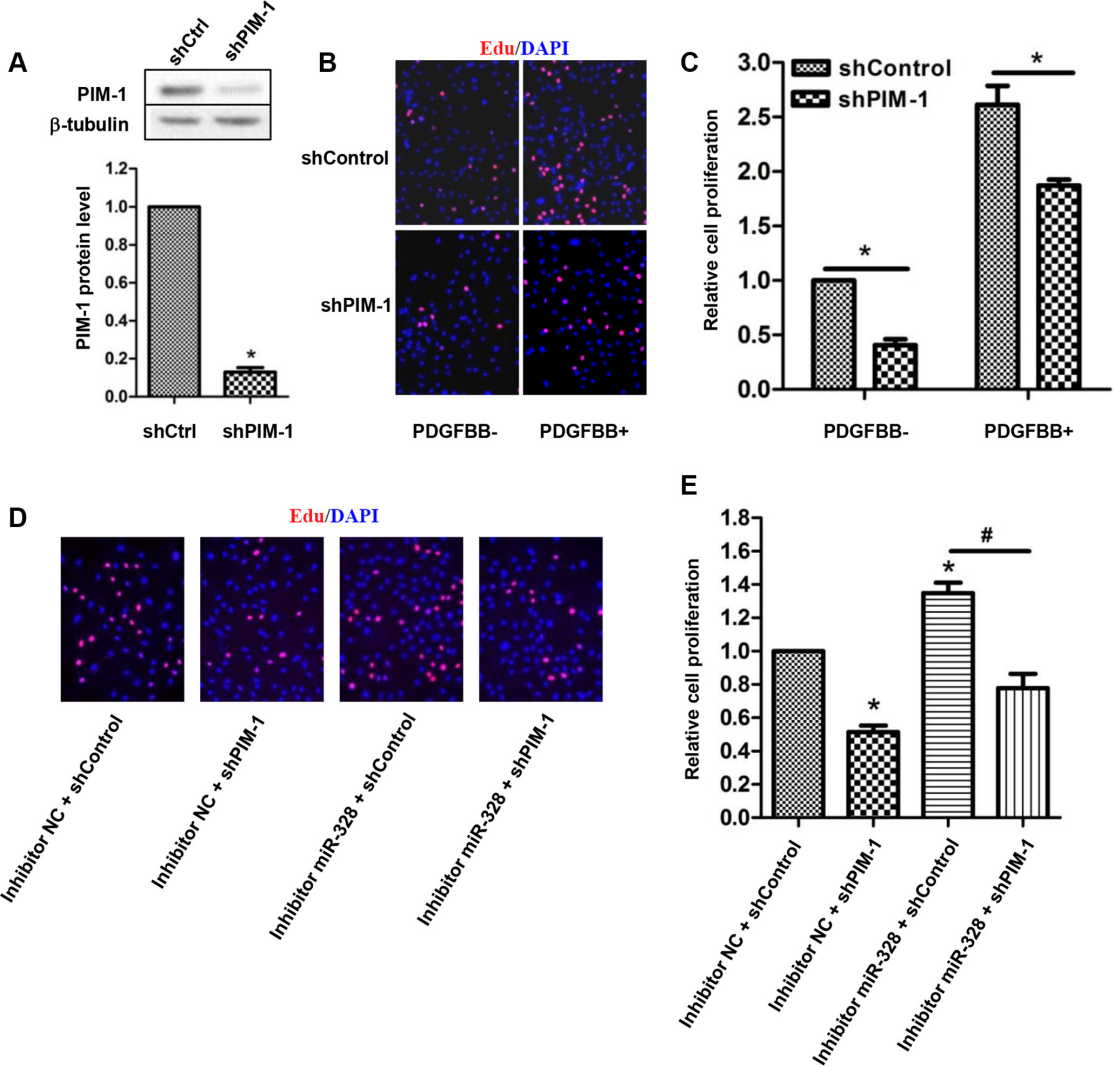
PIM-1 knockdown rescues proliferating PASMCs by inhibition of miR-328 (**A**) Representative western blot of PIM-1 expression (up panel) and the quantification (down panel) in PASMCs infected with shPIM-1 lentivirus. (**B** and **C**) Effect of PIM-1 knockdown on the proliferation of PASMCs stimulated with PDGFBB. (**D** and **E**) Rescue experiments on EdU incorporation assay. PASMCs were transfected with inhibitor miR-328 and then infected with shPIM-1 lentivirus for 48 h, and the cell proliferation was measured. Results are shown as means ± SD of three independent experiments. *p < 0.05 compared to control without PDGFBB treatment (PDGFBB-).

Next, we examined whether PIM-1 knockdown could prevent the increased PASMCs proliferation by suppressing miR-328. To find out this, we firstly investigated the effect of PIM-1 knockdown on PASMCs proliferation. As a result, infection of shPIM-1 lentivirus resulted in a remarkable reduction in PIM-1 protein expression (Figure 5A), which in turn significantly reduced PASMCs proliferation both with and without PDGFBB treatment (Figure 5B and 5C). Subsequently, rescue experiment was carried out to confirm the relation between miR-328 and PIM-1. As shown in Figure 5D and 5E, suppression of miR-328 induced PASMCs proliferation was restored by the knockdown of shPIM-1. Similar results were also observed in PCNA immunoblotting experiments (data not shown). Taken together, these data suggested that miR-328 inhibited PASMCs proliferation through targeting PIM-1.

## PDGFBB-INDUCED MIR-328 DOWNREGULATION NEGATIVELY CORRELATES WITH DNMT1

**Figure 6:**
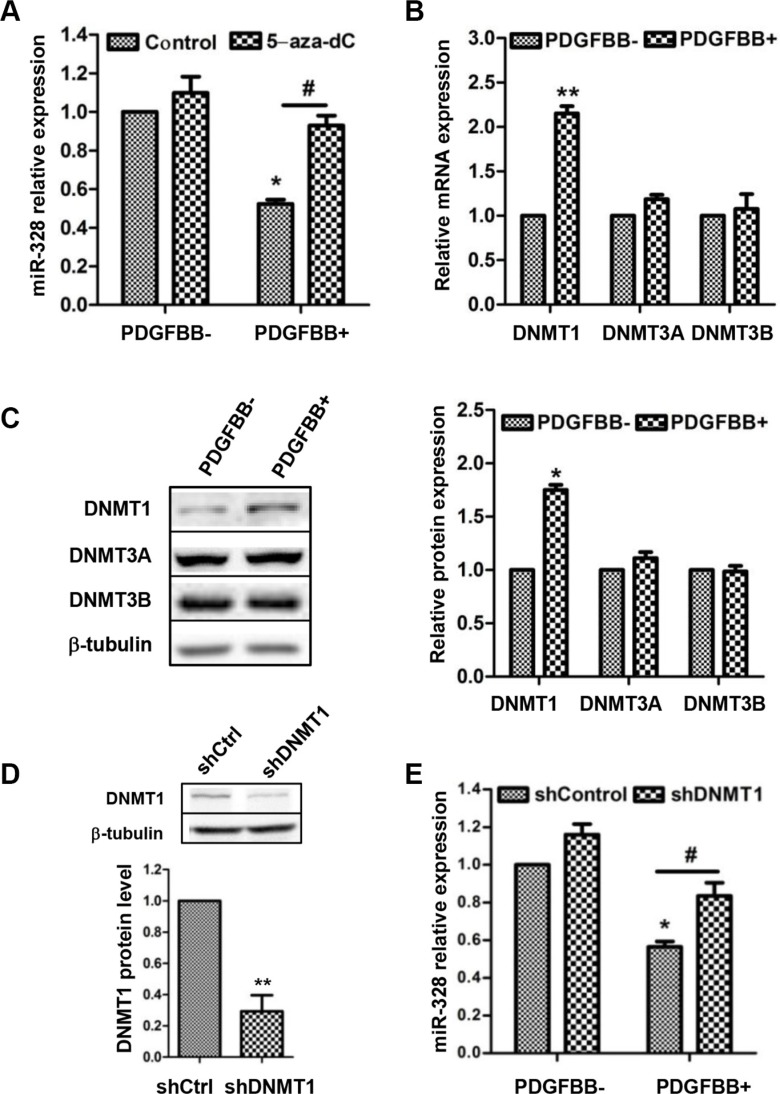
The inhibition of miR-328 by PDGFBB is associated with increased DNMT1 expression PASMCs were pretreated with 5-aza-2′-deoxycytidine (5-aza-dC) in a concentration of 5 mM for 48 h and then stimulated with PDGFBB. MiR-328 expression was determined by qRT–PCR (**A**). The expression of DNMT1, DNMT3A and DNMT3B at mRNA and protein levels were measured in PDGFBB-treated PASMCs using qPCR and western blotting (**B** and **C**). DNMT1 protein level in PASMCs infected with shDNMT1 lentivirus (**D**). After the infection of shDNMT1 levtivirus, miR-328 expression was measured in PASMCs in the absence and presence of PDGFBB (**E**). Results are shown as means ± SD of three independent experiments. *p < 0.05 compared to control without PDGFBB treatment (PDGFBB-), and #*p* < 0.05 compared to control lentivirus with PDGFBB treatment.

The expression of miR-328 was reported to associate with DNA methylation in human placenta [20], thus we hypothesized that similar phenomenon might exist in this work. To test the hypothesis, PASMCs were pretreated with demethylating agent 5-aza-2′-deoxycytidine (5-aza-dC), and then miR-328 expression was determined. As expectation, PDGFBB-induced decrease of miR-328 expression was completely recovered to control level by 5-aza-dC pretreatment (Figure 6A). Since DNA methylation was mainly catalyzed by DNA methylation transferase (DNMT) [21], we therefore measured the expression pattern of three active DNMTs, i.e. DNMT1, DNMT3A and DNMT3B. It turned out that DNMT1 but not DNMT3A and DNMT3B was induced at both mRNA and protein levels in response to PDGFBB (Figure 6B and 6C). Moreover, knockdown of DNMT1 prevented the reduction of miR-328 in PDGFBB-treated PASMCs (Figure 6D and 6E). Taken together, these results indicated that the inhibition of miR-328 by PDGFBB was likely due to the upregulation of DNMT1 and DNA methylation.

### MiR-328 level decreased in serum of CHD-PAH patients

**Figure 7:**
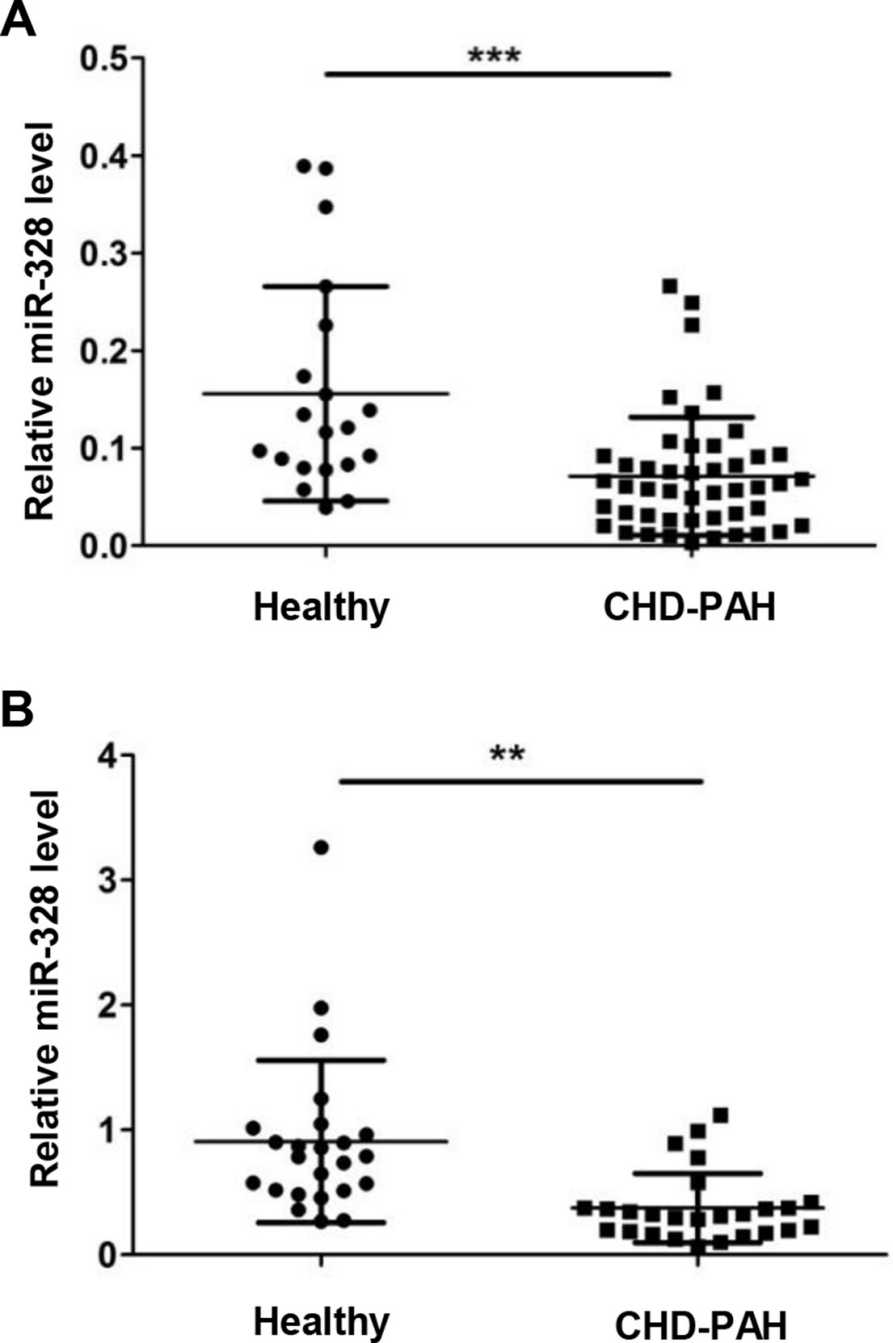
MiR-328 expression is decreased in serum of CHD-PAH patients Serum miR-328 expression was determined in healthy human donors and CHD-PAH patients using qRT-PCR. Relative miR-328 level in 20 healthy versus 49 CHD-PAH patients (**A**), and a second group of 24 healthy versus 26 patients (**B**). Data are shown as means ± SE, **p < 0.01 and ***p < 0.001 compared to healthy donor.

To evaluate whether miR-328 has the potential to be a circulating biomarker, serum miR-328 level was measured in two batches of CHD-PAH patients. The first batch of serum was collected from 20 healthy donors and 49 PAH patients in the Fuwai Hospital. All the subjects are newborns while patients are coupled with ventricular septal defect (VSD) or atrial septal defects (ASD) (Table S1). In comparison with the healthy donors, serum miR-328 expression significantly decreased in CHD-PAH patients (Figure 7A). To verify this result, a second batch of serum from 50 adults consisting of 24 healthy donors and 26 CHD-PAH patients were collected in Sun Yat-Sen Cardiovascular Hospital. Similarly, most of the patients are accompanied by VSD or ASD (Table S2), and the downregulated circulating miR-328 was also observed in CHD-PAH patients (Figure 7B).

## DISCUSSION

**Figure 8:**
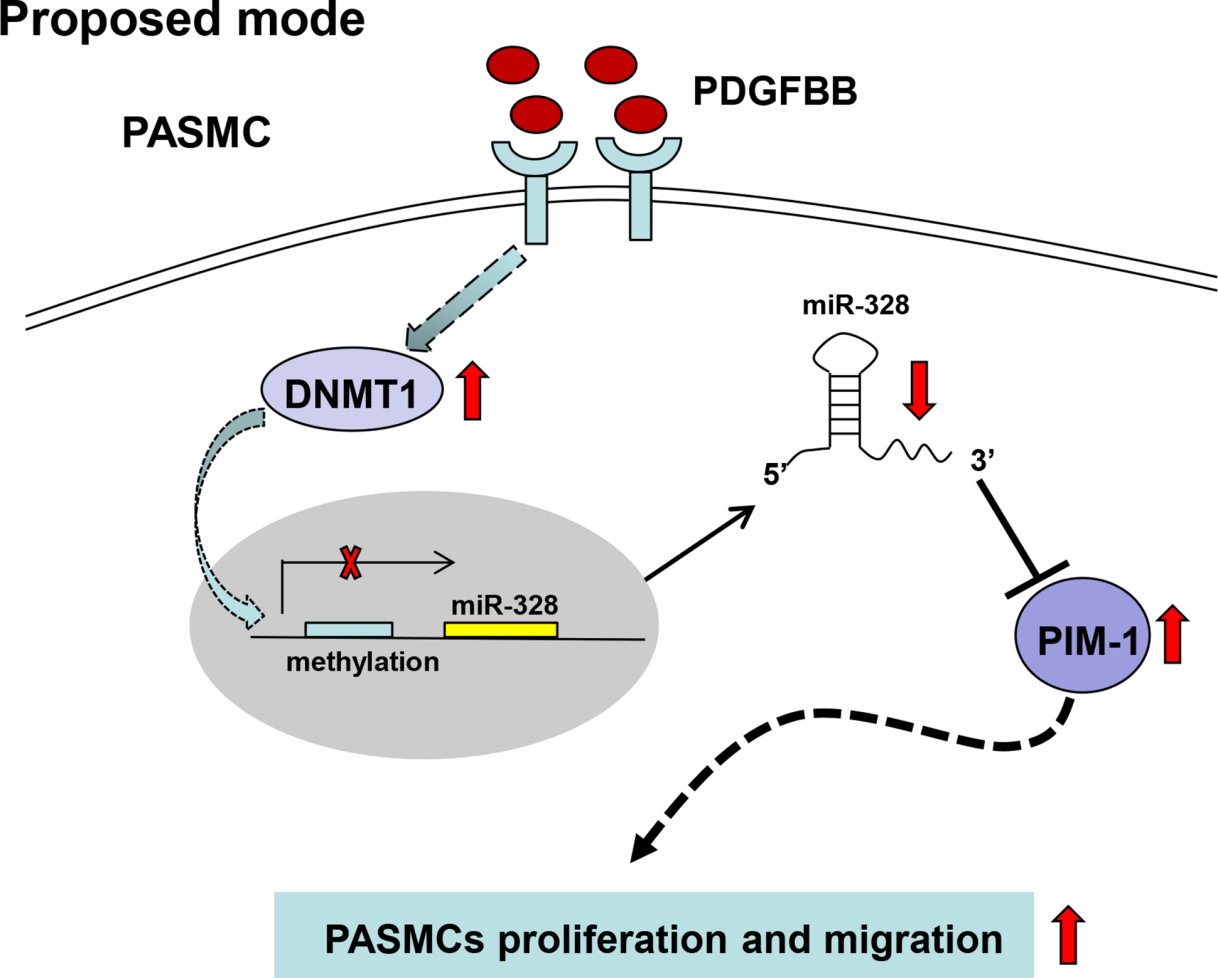
A schematic model that proposed a possible mechanism by which miR-328 was inhibited by PDGF, and the regulatory function of miR-328 through targeting PIM-1 in PASMCs PDGF: platelet derived growth factor; DNMT: DNA methylation transferase; PIM: Ser/Thr-protein kinase-1.

This study is the first one to investigate miRNAs expression profiling at a genomic scale in PDGFBB-stimulated PASMCs, resulting in the recognition of markedly downregulated miR-328. We proposed a possible mechanism that PDGF-induced inhibition of miR-328 was likely due to the increased DNMT1 and DNA methylation, and demonstrated that miR-328 inhibited PASMCs proliferation and migration through targeting PIM-1 (Figure 8). Moreover, this work also suggested that serum miR-328 level could be a potential circulating biomarker for clinical diagnosis in CDH-PAH patients.

Growing studies suggest that miRNA expression can be regulated in PDGF signaling, however, litter is known about their expression profiles in a genome wide view. In this study, we profiled 1,078 miRNAs in PDGFBB treated PASMCs and identified 15 significantly altered miRNAs (Figure 1). In agreement with previous studies [17, 22, 23], the expression of miR-221 and miR-210 were significantly increased in response to PDGFBB (Figure 1C), while the rest of 13 miRNAs are rarely studied. However, some reported miRNAs such as miR-365 [24], miR-136 [18] and miR-599 [25], were unable to screen out in our study. Possible explanations could be that the experimental settings, cell type for experiment and criteria for miRNA screening were different. Most of the previous studies mainly used VSMCs or stem cell for profiling analyses by microarray or deep sequencing [19, 26], whereas we analyzed differentially expressed miRNAs at genomic scale in PASMCs using a sensitive qPCR-based miRNA assay [27].

We noted that, miR-328 expression was specifically inhibited by PDGFBB rather than other growth factors in a dose- and time-dependent manner (Figure 1E–1G), suggesting a major role of miR-328 in PDGFBB associated cell function [19, 24]. Moreover, it has been found that miR-328 was majorly localized in PASMCs, and it played a significant role in pulmonary artery remodeling and hypoxic pulmonary hypertension by promoting PASMCs apoptosis [28]. Therefore, regarding its crucial function in PASMCs, miR-328 was selected for further study thereafter.

Subsequent functional analyses revealed that miR-328 is tightly associated with PASMCs proliferation and migration in PDGFBB pathway, as if the suppression of miR-328 by PDGFBB promoted PASMCs proliferation and migration, while overexpression of miR-328 displayed an opposite function (Figure 2 and Figure 3). Thus, these findings indicated that miR-328 indeed play a regulatory role in abnormal proliferation and migration of PASMCs stimulated by PDGFBB.

Next, we demonstrated that miR-328 directly interacted with the 3′-UTR of *PIM-1*, and negatively regulate PIM-1 expression in PASMCs (Figure 4). PIM-1 is a proto-oncoprotein that belongs to the serine/threonine protein kinase family. In addition to its pivotal role in tumorigenesis [29, 30], PIM-1 has also been implicated in vascular diseases [31, 32]. Moreover, the PIM-1 plasma level has been suggested as PAH biomarker [33]. In this work, the expression of PIM-1 showed a significant increase in response to PDGFBB (Figure S1), agreeing with the study of Willert *et al.* [34]. We also found that knockdown of PIM-1 inhibited PASMCs proliferation (Figure 5B and 5C), which is coordinated with the observation that overexpressing miR-328 could suppress the proliferation of PASMCs (Figure 2). Most importantly, increased proliferation of PASMCs by suppressing miR-328 could be recovered by PIM-1 knockdown (Figure 5D and 5E). Therefore, these results clearly demonstrated that miR-328 inhibited PDGFBB induced PASMCs proliferation through targeting PIM-1. Nevertheless, more in-depth study will be performed in future to confirm the role of miR-328 in animal models.

It is interesting to find that the inhibition of miR-328 by PDGFBB was completely blocked by the pretreatment of DNMT inhibitor 5-aza-dC (Figure 6A), implying the PDGFBB-induced downregulation of miR-328 is likely associated with DNA methylation. Previous study has shown that the DNA methylation level of CpG sites in the 5′-flanking region of miR-328 was negatively correlate with its expression [20]. To further explore which DNMT is responsible for the DNA methylation, we measured the expression of three active DNMTs. It was found that the expression of DNMT1 was significantly upregulated by PDGFBB (Figure 6B and 6C), which is consistent with the result in rat airway SMCs [35]. DNMT1 is the most abundant and considered to be a key methyltransferase for the maintenance of DNA methylation [21, 35]. Consequently, we assumed PDGF induced DNMT1 catalyzing DNA methylation is likely contribute to the decrease of miR-328, leading to the upregulation of PIM-1 and PASMCs aberrant proliferation and migration (Figure 8). A further support to this scenario was that knockdown of DNMT1 significantly restored the decrease of miR-328 (Figure 6D and 6E) and the increase of PIM-1 by PDGFBB (Figure S2). Nevertheless, future experiments are required to study the exact position of DNA methylation in PDGF signaling.

Dysregulation of circulating miR-328 has been indicated as predictive biomarkers for various diseases. For instance, increased circulation miR-328 level has a potential for the early diagnosis of non-small cell lung cancer [36] and acute myocardial infarction [37]. By contrast, the reduction of circulating miR-328 could be an indicator for the poor prognosis in acute myeloid leukemia patients [38]. In patients with pulmonary hypertension, circulating miRNAs are speculated to be released from the damaged lungs or vessels [39]. In this work, we found serum miR-328 level was significantly decreased in the two batches of CHD-PAH patients (Figure 7), indicating a potential value of circulating miR-328 as a biomarker in CHD-PAH. However, our results are based on a limited number of clinical specimens, and additional study will be needed for further validation in a larger cohort and with early onset of PAH.

## MATERIALS AND METHODS

### Cell culture and treatment

Human embryonic kidney (HEK) 293 cells were obtained from American Type Culture Collection (ATCC, Manassas, USA) and maintained in Dulbecco's modified Eagle's medium (DMEM) supplemented with 10% fetal bovine serum (FBS). Human pulmonary arterial smooth muscle cells (PASMCs) were purchased from Sciencell (San Diego, CA, USA) and cultured in smooth muscle cell medium (SMCM) as previously described [40]. Rat PASMCs were isolated and cultured in DMEM supplemented with 10% FBS as reported by Zeng *et.al*. [15]. PASMCs at passage 3 or 4 were used for experiments, and the contractile phenotype was confirmed by the determination of contractile marker genes expression, such as a-SMA and SM22 [16]. Growth factors including PDGFBB, AngII, TGF-b, IGF, VEGF, ET-1, PDGFAA and FGF were obtained from R&D Systems. For the growth factors treatment, PASMCs were starved for 12h in starvation condition (0.2% FBS) and then stimulated with the growth factors at concentration as indicated.

### Vectors construction

Lentiviral vector inserting short hairpin RNA (shRNA) targeted to PIM-1 or DNMT1 were constructed based on pLVX-hU6. The shRNA sequences were prepared by two primers annealing. For shPIM-1 primer 1: 5′-ACCGTGCAAGACCTCTTCGACTT CACTCGAGT GAAGTCGAAGAGGTCTTGCA-3′, primer 2: 5′-AAA ATGCAAG ACCTCTTCGACTTCACTCGAGTGAAG TCGAAGAGGTCTTGCA-3′; for shDNMT1, primer 1: 5′-AGCGAGGAGAACGGTGCTCATGCTTATAGTGAA GC CACAGATGTATAAGCATGAGCACCGTTCTCC-3′, primer 2: 5′-GGCAGGGA GAACGGTGCTCATGCT TATACATCTGTGGCTTCACTATAAGCATGAGCAC C GTTCTCC -3′. For the luciferase plasmid construction, the 3′-UTR of PIM-1 was amplified from human genomic DNA and cloned into pmirGLO dual-luciferase vector (Promega, Madison, WI, USA). To construct mutational 3′-UTR report vector, the region that base-paired with miR-328 seeding sequences were mutated by site-directed mutagenesis. The primer sequences for PIM-1 3′-UTR WT forward primer: 5′-CG GAATTCCAGCCTTTCTGGCAGGTCCTCCC CT-3′ and reverse primer: 5′-CCGC TCGAGGGAAGGC ACACCATCCAGAACTGCT-3′; for PIM-1 3′-UTR mutation forward primer: 5′-GGTGCCATGGAAGAGGC TACTCCCGGTA ACGCTGAGCCACCTGCCCT-3′ and reverse primer: 5′-AGGGCAGGTGGCTCAGCGTTA CCGGGAGTAGCCTCTTCCATGGCACC-3′. All the constructs were confirmed by DNA sequencing.

### miRNA transfection and lentivirus infection

Chemically synthesized mimic and inhibitor miR-328 for overexpression and suppress of endogenous mature miR-328 expression, and the corresponding miRNA negative control (mimic and inhibitor NC) were provided by Ruibobio (Guangzhou, China). The miRNA mimic and inhibitor were transfected in a final concentration of 20 nM and 50 nM, respectively, using K2 transfection reagent (Biontex, Germany) as recommended by the manufacturer. The generation of high titer lentivirus and the PASMCs infection procedures were performed as previously described [40].

### Cell proliferation and migration assays

Cell proliferation was measured by EdU incorporation and PCNA immunoblotting. EdU labeling was carried out using the EdU Assay Kit (Ribobio, Guangzhou, China) according to the manufacturer's instruction. The stained cells were examined using a fluorescent-inverted microscope. Cell proliferation rate was calculated as the number of cells with EdU staining divided by the number of cells stained with DAPI. PCNA protein expression was determined as described in western blotting.

Cell migration was determined by wound-healing assay as reported by Yang *et. al*. [41]. In brief, transfected cells were maintained in starvation condition for 12 h and a scratch was made in the cell layer. Following the photograph of initial image, the cell migration was determined by measuring the decreased width of the scratch after 24 h of PDGFBB stimulation.

### Quantification of mRNA and miRNA expression

Total RNA was extracted with RNAiso Plus (Takara, Dalian, China) and quantified using the NanoDrop 2000c Spectrophotometer (Thermo Fisher Scientific, Wilmington, DE). For mRNA expression detection, first-strand cDNA was synthesized from 1 mg of total RNA treated by DNase using oligo(dT) [18] plus random hexamer primers with M-MLV Reverse Transcriptase (Invitrogen). The quantitative PCR experiments were performed on Step-One plus real-time PCR System (Applied Biosystems) using SYBR green-I Master PCR Mix with gene specific primers. The β-actin was used as reference gene for normalization. Primers used for mRNA determination were listed as follows, *PIM-1* forward primer: 5′-GCCCTCCTTTGAAGAAATCCAGAAC-3′ and reverse primer: 5′-ACTGAGTCTGTGAGGGGCA AAGCGG-3′; *DNMT1* forward primer: 5′-CAATGAGGCA CTGTCCGTCT-3′ and reverse primer: 5′-CCA GGTTC TTGCCATTAACACCA-3′; *DNMT3A* forward primer: 5′-TACCACCAGG TCAAACTCCATAAAG-3′ and reverse primer: 5′-TCTGGGTGCTGAACTTCTCT CCGTC-3′; *DNMT3B* forward primer: 5′-ATGGCAA GGATGACGTTCTGTGGT G-3′ and reverse primer: 5′-GCTAAGGAAGGGGACAGGTGAGGTG-3′; *β-actin* forward primer: 5′-AAAGACCTGTACGCCAACAC-3′ and reverse primer: 5′-GTC ATACTCCTGCTTGCTGAT-3′.

The miRNA expression was determined using S-Poly (T) Plus real-time PCR method as detailed in our previous work [27, 42], and snoRNA-202 was used reference gene. The primer sequences for miRNA determination were, *miR-328* RT primer: 5′-GTGCAGGGTC CGAGGTCAGAGCCACCTGGGCAATTTTTT TTTTT ACGGAA-3′ and forward primer: 5′-CCCACCCTGGCCCTCTCTGC-3′; sno202 RT primer: 5′-GTGCAGGGTCCGAGGTCAGA GCCACCTGGGCAATTTTTTTTT TTCATCA G-3′ and forward primer 5′-GTACTTTT GAACCCTTTTCCAT-3′. The relative expression of mRNA and miRNA was calculated using the 2^−ΔΔCt^ method.

### Western blotting

Total proteins were extracted using ice-cold RIPA buffer (50 mM Tris-HCl, pH 7.5; 150 mM NaCl; 1% NP-40; 0.25% sodium deoxycholate, 1mM EDTA), supplemented with a protease inhibitor cocktail. The samples were homogenized and centrifuged for 10 min at 12000 g at 4°C. The resulting supernatant was collected and used for protein concentration determination by BCA assay protein kit (Thermo scientific). Equal amounts of proteins (30 μg) were electrophoresed on a sodium dodecyl sulfate (SDS) polyacrylamide gel and transferred to a polyvinylidene fluoride (PVDF) membrane (Millipore, Billerica, MA, USA). The membranes were blocked for 2 h at room temperature in Tris-Buffered saline with 0.1% Tween 20 (TBST), containing 5% non-fatted dry milk (blocking solution). After that, the membrane was incubated with antibodies against PIM-1 (Abcam, USA), PCNA (Sanying, Wuhan, China), DNMT1, DNMT3A, DNMT3B and β-tubulin (Santa Cruz Biotechnology, Santa Cruz, CA) overnight at 4°C. After 3 × 10 min washes in TBST solution, the membrane was incubated for 1 h at room temperature with horseradish peroxidase-conjugated secondary antibody (Jackson Immuno-Research, West Grove, PA). The protein bands were visualized using the SuperSignal chemiluminescent detection module (Pierce).

### Luciferase reporter assay

For luciferase activity measurement, HEK293 cells were seeded in 48-well plates and co-transfected with luciferase reporter plasmid together with either mimic miR-328 or mimic NC. The cells were lysed for luciferase activity measurement using a Lumat LB9508 luminometer (Berthold, Bad Wildbad, Germany) after 48 h of transfection. The firefly luciferase activity expressed in the same vector was used as an internal control for normalization.

### Collection of human serum sample

Two batches of serum samples were collected from healthy participants and CHD-PAH patients at the Fuwai Hospital (Beijing, China) and Sun Yat-Sen Cardiovascular Hospital (Shenzhen, China), respectively. The blood samples were centrifuged at 3000g for 10 min at 4°C and stored at −80°C until use. The study was approved by the ethics committee of the two Hospitals. All subjects who participated in the study provided written informed consent.

### Statistical analysis

Statistical analyses were carried out using the SPSS version 13.0 software 261 package (SPSS, Chicago, IL, USA) for Windows. All data are presented as mean values ± standard deviation (SD) of three experiments. When only two groups were compared, the statistical differences were evaluated with the double-sided Student's *t*-test. Significant differences between groups were analyzed by one-way analysis of variance (ANOVA), taking *p* value less than 0.05 (*or ^#^ < 0.05,** < 0.01, *** < 0.001) as a significant difference.

## CONCLUSION

In summary, our results demonstrated that PDGFBB-caused downregulation of miR-328 is likely attributed to the upregulation of DNMT1 and DNA methylation, while miR-328 inhibits PASMCs proliferation and migration by targeting PIM-1. We also highlight the potential of circulating miR-328 serving as a biomarker for the diagnosis of CHD-PAH.

## SUPPLEMENTARY MATERIALS




