# Supplementary material for: MiR-328 targeting PIM-1 inhibits proliferation and migration of pulmonary arterial smooth muscle cells in PDGFBB signaling pathway
Source: Oncotarget. 2016 Jul 19;7(34):54998–5011. doi: 10.18632/oncotarget.10714 (PMC5342397; doi:10.18632/oncotarget.10714)
Supplement: Supplementary file 1 [file oncotarget-07-54998-s001.pdf]

MiR-328 targeting PIM-1 inhibits proliferation and migration of pulmonary arterial smooth muscle cells in PDGFBB signaling pathway

Supplementary Materials

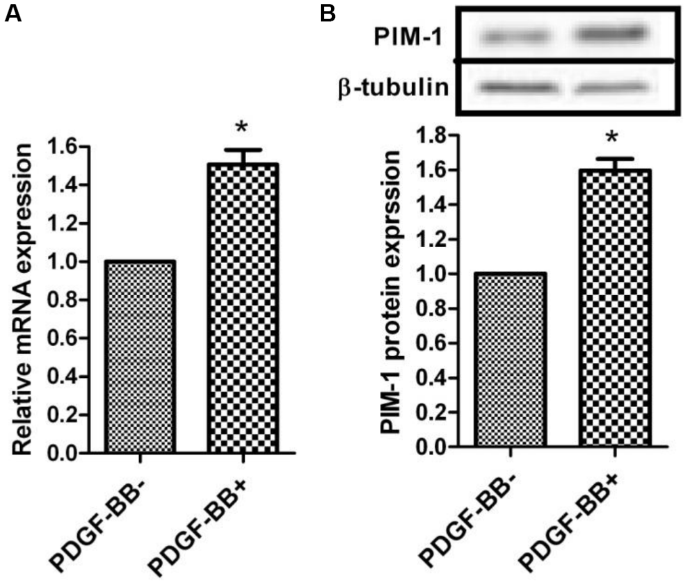

Supplementary Figure S1: PDGFBB induces the expression of PIM-1. PASMCs were treated with PDGFBB (20 ng/mL) for 48 h and the cells were collected. The expression of PIM-1 was measured at mRNA level using qRT-PCR (**A**) and protein level by western blotting (**B**). Data are shown as means ± SD of three independent experiments. *$P < 0.05$ compared to control without PDGFBB treatment (PDGFBB-).

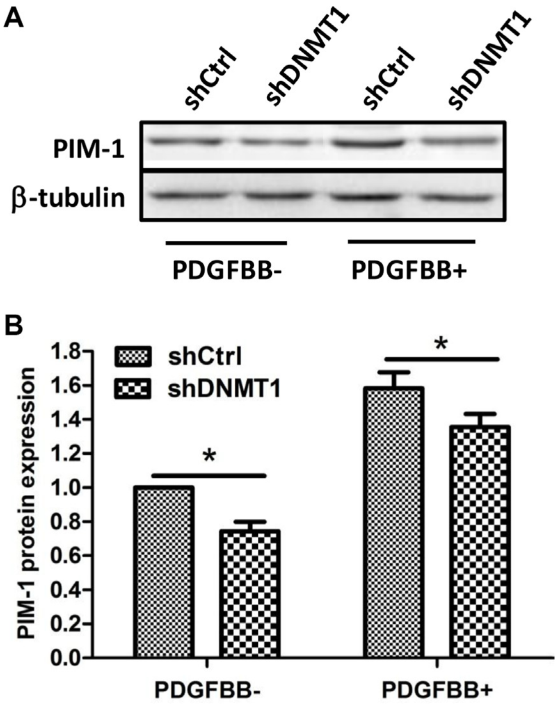

Supplementary Figure S2: Knockdown of DNMT1 inhibits PIM-1 expression. PASMCs were infected with shDNMT1 lentivirus for 72 h and then stimulated with or without PDGFBB (20 ng/mL). PIM-1 protein expression was measured by western blotting. Representative results of immunoblots of PIM-1 (**A**) and protein quantifications (**B**) are presented. Data are shown as means ± SD of three independent experiments. $^*P < 0.05$ compared to control without PDGFBB treatment (PDGFBB-).

Supplementary Table S1: The information of the first batch of CHD-PAH patients and healthy donors from the fuwai hospital. See Supplementary_Table_S1

Supplementary Table S2: The information of the second batch of CHD-PAH patients and healthy donors from the Sun Yat-Sen cardiovascular hospital

CHD-PAH patients				
NO.	Gender	Age(years)	Diagnosis	Remarks
1	Male	50	MSI, Severe PAH	B-scan ultrasonography,66 mmHg,hemolysis
2	Male	30	IPAH	
3	Male	35	ASD, Postoperative	
4	Male	60	MS, Moderate PAH	
5	Male	42	VSD, Moderate-Severe PAH	
6	Male	32	VSD	
7	Male	5	ASD	
8	Male	37	ASD	
9	Male	42	VSD, Moderate-Severe PAH	Cardiac Catheterization,91/46 mmHg
10	Female	25	PDA, Severe PAH	Cardiac Catheterization,105/68 mmHg
11	Female	35	IPAH, Severe	B-scan ultrasonography,106/56 mmHg
12	Female	27	ASD, Mild PAH	B-scan ultrasonography, 34 mmHg
13	Female	29	ASD, Severe PAH	B-scan ultrasonography, 93/160 mmHg
14	Female	24	ASD, Mild PAH	B-scan ultrasonography, 34 mmHg
15	Female	18	ASD, Mild PAH	B-scan ultrasonography, 37 mmHg
16	Female	20	VSD, Postoperative, Severe PAH	
17	Female	31	VSD, Severe PAH	
18	Female	33	ASD, Postoperative PAH	
19	Female	37	ASD, Moderate-Severe PAH	
20	Female	42	MSI	B-scan ultrasonography, 42 mmHg
21	Female	38	MSI	B-scan ultrasonography, 50 mmHg
22	Female	45	ASD	B-scan ultrasonography, 42 mmHg
23	Female	45	ASD	B-scan ultrasonography, 39 mmHg
24	Female	32	ASD	B-scan ultrasonography, 38 mmHg
25	Female	64	MSI	B-scan ultrasonography, 35 mmHg
26	Female	30	ASD	B-scan ultrasonography, 66 mmHg

Healthy donors					
NO.	Gender	Age(years)	NO	Gender	Age(years)
1	Male	35	13	Female	24
2	Male	58	14	Female	16
3	Male	42	15	Female	21
4	Male	32	16	Female	31
5	Male	26	17	Female	33
6	Male	37	18	Female	37
7	Male	40	19	Female	43
8	Male	56	20	Female	38
9	Female	25	21	Female	32
10	Female	35	22	Female	42
11	Female	27	23	Female	22
12	Female	29	24	Female	28

Notes: ASD, atrial septal defect; MS, mitral stenosis; VSD, ventricular septal defect; PDA, Patent Ductus Arteriosus; IPAH, Idiopathic Pulmonary Arterial Hypertension; MSI, mitral stenosis and insufficiency.
