# Supplementary material for: MiR-328 targeting PIM-1 inhibits proliferation and migration of pulmonary arterial smooth muscle cells in PDGFBB signaling pathway
Source: Oncotarget. 2016 Jul 19;7(34):54998–5011. doi: 10.18632/oncotarget.10714 (PMC5342397; doi:10.18632/oncotarget.10714)
Supplement: Supplementary file 2 [file oncotarget-07-54998-s002.docx]

**Supplementary Table S1: The information of the first batch of CHD-PAH patients and healthy donors from the fuwai hospital**

| **CHD-PAH patients** | | | | |
| --- | --- | --- | --- | --- |
| NO | Patient’s ID | Gender | Age | Diagnosis |
| 1 | 754937 | 女 | 1 years + 2 months | ASD, Mild PAH |
| 2 | 757064 | 女 | 11 months | ASD, Mild PAH |
| 3 | 757808 | 男 | 2 years + 6 months | ASD, Mild PAH |
| 4 | 757833 | 女 | 1 years + 3 months | ASD, Moderate PAH |
| 5 | 758474 | 男 | 4 years + 7 months | ASD, Severe PAH |
| 6 | 759373 | 女 | 9 months | VSD, Mild PAH |
| 7 | 761435 | 女 | 7 months | VSD, Mild PAH |
| 8 | 771706 | 男 | 3 years + 1 months | VSD, Severe PAH |
| 9 | 785003 | 男 | 5 months | ASD, Mild PAH |
| 10 | 789888 | 女 | 5 months | VSD, Mild PAH |
| 11 | 794091 | 女 | 3 years | ASD |
| 12 | 795174 | 男 | 8 months | VSD, CHD |
| 13 | 795633 | 女 | 3 years | ASD |
| 14 | 795829 | 女 | 10months | VSD, Postoperative |
| 15 | 795836 | 男 | 5 months | ASD, Postoperative |
| 16 | 795877 | 男 | 2 years | ASD, Postoperative |
| 17 | 796006 | 男 | 3 years | VSD, Postoperative |
| 18 | 796026 | 男 | 1 years | ASD |
| 19 | 796058 | 男 | 2 years + 9 months | ASD, Mild PAH |
| 20 | 796085 | 男 | 2 years | ASD, Postoperative |
| 21 | 796320 | 女 | 3 years | VSD |
| 22 | 797712 | 男 | 2 years | VSD, CHD |
| 23 | 797743 | 男 | 1 years | ASD, Mild PAH |
| 24 | 799233 | 女 | 4 months | VSD, Severe PAH |
| 25 | 804178 | 男 | 3 months | ASD, Mild PAH |
| 26 | 804421 | 男 | 1 years + 7 months | ASD, Mild PAH |
| 27 | 804626 | 女 | 3 years | ASD, Severe PAH |
| 28 | 804694 | 女 | 6 months | VSD, Moderate PAH |
| 29 | 805218 | 女 | 1 years | VSD, Severe PAH |
| 30 | 805718 | 女 | 9 months | VSD, Moderate PAH |
| 31 | 805926 | 男 | 4 years + 6 months | VSD, Severe PAH |
| 32 | 805929 | 男 | 1 years + 3 months | VSD, Mild PAH |
| 33 | 806394 | 男 | 6 months | ASD, Severe PAH |
| 34 | 808020 | 女 | 9 months | ASD, Mild PAH |
| 35 | 808091 | 男 | 6 months | ASD, Severe PAH |
| 36 | 810394 | 男 | 1 years | ASD, Moderate PAH |
| 37 | 811536 | 男 | 9 months | VSD, Severe PAH |
| 38 | 811589 | 女 | 11 months | VSD, Mild PAH |
| 39 | 812097 | 女 | 6 months | VSD, Mild PAH |
| 40 | 813105 | 男 | 5 months | VSD, Severe PAH |
| 41 | 813172 | 女 | 6 months | VSD, Moderate PAH |
| 42 | 813183 | 男 | 4 months | ASD, Mild PAH |
| 43 | 813925 | 女 | 11 months | VSD, Mild PAH |
| 44 | 814453 | 女 | 3 years + 11 months | ASD, Severe PAH |
| 45 | 815113 | 女 | 1 years + 5 months | VSD, Severe PAH |
| 46 | 815125 | 男 | 1 years + 11 months | ASD, Mild PAH |
| 47 | 815364 | 男 | 10 years | VSD, Severe PAH |
| 48 | 816647 | 男 | 11 months | VSD, Severe PAH |
| 49 | 816805 | 男 | 5 months | ASD, Moderate PAH |

| Healthy donors | | |
| --- | --- | --- |
| NO | Gender | Age |
| 1 | 男 | 9 months |
| 2 | 男 | 1 years |
| 3 | 男 | 3 years |
| 4 | 女 | 1 years |
| 5 | 男 | 28 days |
| 6 | 女 | 1 years |
| 7 | 男 | 2 years |
| 8 | 男 | 2 years |
| 9 | 男 | 3 months + 23 days |
| 10 | 女 | 3 years |
| 11 | 男 | 16 hours |
| 12 | 女 | 5 months + 9 days |
| 13 | 女 | 3 days |
| 14 | 女 | 3 years |
| 15 | 男 | 6 days |
| 16 | 女 | 7 days |
| 17 | 男 | 3 months + 23days |
| 18 | 女 | 15 hours |
| 19 | 女 | 15 hours |
| 20 | 女 | 17 hours |

Notes: ASD, atrial septal defect; VSD, ventricular septal defect; CHD, congenital heart disease; PAH, Pulmonary Arterial Hypertension.
